# The influencing factors of postoperative sleep disturbance in elderly patients undergoing transurethral prostate surgery and the construction and validation of a machine learning prediction model

**DOI:** 10.3389/fmed.2026.1962940

**Published:** 2026-10-21

**Authors:** Jingxian Huang, Huageng Huang, Yuhua Zhou, Yubo Xie

**Affiliations:** 1 Department of Anesthesiology, The First Affiliated Hospital of Guangxi Medical University, Nanning, China; 2 Department of Anesthesiology, The Third Affiliated Hospital of Guangxi Medical University, Nanning, China; 3 Department of Anesthesiology, Sichuan Clinical Research Center for Cancer, Sichuan Cancer Hospital & Institute, Sichuan Cancer Center, University of Electronic Science and Technology of China, Chengdu, China

**Keywords:** elderly patients, machine learning, postoperative sleep disturbance, prediction model, transurethral prostate surgery

## Abstract

**Background:**

Postoperative sleep disturbance (POSD) is a common complication in elderly patients undergoing transurethral prostate surgery, which impairs recovery and increases disease burden. To date, prediction models for POSD in this population, particularly those based on machine learning techniques, remain scarce.

**Objective:**

To investigate the incidence and influencing factors of POSD in elderly patients undergoing transurethral prostate surgery, and to construct prediction models based on conventional logistic regression and seven machine learning algorithms.

**Methods:**

A total of 563 elderly patients who underwent elective transurethral prostate surgery at our hospital between March 2024 and December 2025 were prospectively enrolled and randomly divided into a training set (*n* = 338) and a validation set (*n* = 225) at a ratio of 6:4. LASSO regression (with lambda.1se) was used to screen predictive variables, and multivariate logistic regression to identify independent risk factors. Logistic regression, random forest (RF), extreme gradient boosting (XGBoost), naive Bayes, decision tree, support vector machine, k-nearest neighbors, and artificial neural network models were constructed. Model performance was evaluated using area under the receiver operating characteristic curve (AUC), accuracy, calibration curves, and decision curve analysis. SHAP (SHapley Additive exPlanations) was employed for interpretability analysis of the optimal model.

**Results:**

The incidence of POSD was 62.2%. Independent risk factors included preoperative anxiety (Hospital Anxiety and Depression Scale-Anxiety [HADS-A] score), nocturia frequency (≥3 times/night), elevated systemic immune-inflammation index (SII), preoperative noise score, and moderate-to-severe cough-related pain (*P* < 0.05). The random forest model demonstrated the best overall performance, with an AUC of 0.926 (95% CI: 0.89–0.96) in the training set and 0.964 (95% CI: 0.93–0.99) in the validation set, and exhibited superior calibration and clinical utility compared with the other models. SHAP analysis revealed that the HADS-A score contributed the most to the predictions.

**Conclusions:**

Preoperative anxiety, increased nocturia frequency (≥3 times/night), elevated SII, high noise score, and moderate-to-severe cough-related pain are independent risk factors for POSD in elderly patients undergoing transurethral prostate surgery. The random forest prediction model exhibits optimal performance and can be used for early clinical risk identification and individualized intervention.

**Clinical trial registration:**

Chinese Clinical Trial Registry ChiCTR2600124377.

## 1 Introduction

As the global population ages, the proportion of elderly surgical patients continues to rise, and the quality of perioperative management has attracted considerable attention (1). Postoperative sleep disturbance (POSD) is a common perioperative complication in elderly patients; it can increase pain sensitivity, induce postoperative delirium and cardiovascular events, prolong hospital stay, and elevate all-cause mortality, thereby significantly increasing the disease burden (2–4).

The incidence of POSD has been reported to range from 15% to 72%, with a higher rate among elderly patients (5, 6). Transurethral prostate surgery is the primary procedure for relieving bladder outlet obstruction caused by prostate diseases. Elderly patients undergoing this procedure often experience long-term preoperative nocturia, resulting in a “baseline sleep debt.” Postoperatively, factors such as catheter-related bladder discomfort, bladder spasms,continuous bladder irrigation, surgical trauma, and environmental disturbances further elevate the risk of POSD (7). Elucidating the influencing factors is of significant clinical value for optimizing perioperative management and improving patient outcomes.

Clinical prediction models can integrate multidimensional information to achieve early risk stratification (8). Currently, most prediction models for POSD are based on conventional logistic regression, an approach that relies on linear assumptions (9). However, the occurrence of POSD involves complex non-linear interactions among demographic, clinical, and environmental factors (10). Machine learning can automatically capture non-linear relationships and high-order interactions, offering unique advantages in this field (11). Nevertheless, research on machine-learning-based POSD prediction models specifically targeting elderly patients undergoing transurethral prostate surgery remains lacking. This study aims to investigate the incidence and influencing factors of POSD in this population and to construct and validate prediction models using logistic regression and seven mainstream machine learning algorithms, thereby providing a quantitative decision-making basis for early warning, risk stratification, and individualized intervention.

## 2 Materials and methods

### 2.1 General information

We prospectively enrolled 563 elderly patients who underwent elective transurethral prostate surgery under general anesthesia at our hospital between March 2024 and December 2025. The patients were randomly allocated into a training set (*n* = 338) and a validation set (*n* = 225) at a ratio of 6:4. Data from the training set were used for analysis of influencing factors and model development, while data from the validation set were used for internal validation.

Inclusion criteria: (1) elective transurethral prostate surgery under general anesthesia; (2) age 65–90 years; (3) American Society of Anesthesiologists (ASA) physical status I-III; (4) ability to complete questionnaires. Exclusion criteria: (1) patient or family refusal to participate in the study; (2) preoperative Richards-Campbell Sleep Questionnaire (RCSQ) score ≤50 and/or Athens Insomnia Scale (AIS) score ≥6; (3) use of hypnotics or sedatives within the past month; (4) recent history of substance abuse (including but not limited to opioids, benzodiazepines, etc.); (5) severe cognitive dysfunction (e.g., dementia, Alzheimer's disease) or communication disorders; (6) severe cardiopulmonary disease, severe renal impairment requiring dialysis, or severe hepatic impairment of Child-Pugh class C; (7) need for postoperative admission to the intensive care unit.

The study adhered to the ethical principles of the Declaration of Helsinki, was approved by the hospital ethics committee (No. 2024-K222-01), and was registered in the Chinese Clinical Trial Registry (ChiCTR2600124377). Written informed consent was obtained from all patients.

### 2.2 Outcome variables

The primary outcome was perioperative sleep disturbance (POSD). POSD was defined as a Richards-Campbell Sleep Questionnaire (RCSQ) score <50 points and/or an Athens Insomnia Scale (AIS) score ≥6 points at any assessment point on postoperative day 1 or day 3.

The RCSQ consists of five sleep-related items (sleep depth, difficulty falling asleep, number of awakenings, difficulty returning to sleep, and overall sleep quality) and one separate item on noise disturbance. The first five items are scored using a visual analogue scale (0–100 points), with a mean score <50 indicating sleep disturbance (12). The sixth item assesses the impact of noise on sleep, with higher scores indicating greater disturbance. This item was used to evaluate noise levels in the present study. The AIS comprises eight items (sleep onset, nocturnal awakenings, early morning awakening, total sleep time, sleep quality, daytime mood, daytime physical function, and daytime sleepiness). Each item is scored from 0 to 3 points, and a total score ≥6 points is defined as sleep disturbance (13).

### 2.3 Potential predictors

Based on literature review, clinical experience, and pilot experiments, 44 variables were collected, including demographic data (age, sex, smoking, alcohol, medical history), clinical data (surgery time, anesthesia time, extubation time), laboratory results (blood routine, C-reactive protein, systemic immune-inflammation index [SII]), and preoperative questionnaire results (numerical pain rating scale, Hospital Anxiety and Depression Scale [HADS], noise score). The pain visual analogue scale (VAS) was scored as: 1–3 mild, 4–6 moderate, 7–10 severe. Moderate-to-severe preoperative cough-related pain was defined as a VAS on movement score >3 points. The HADS consists of an anxiety subscale (HADS-A) and a depression subscale (HADS-D); a score ≥8 on either subscale indicates anxiety or depression (14). SII was calculated as (platelet count×neutrophil count)/lymphocyte count.

### 2.4 Sample size estimation

Based on previous studies and pilot experiments, the estimated incidence of POSD was approximately 70%. With 20 candidate variables planned for inclusion, the minimum required sample size was calculated using the 10 events per variable (EPV) principle as 10 × 20/0.7≈286 cases. After accounting for a potential 10% missing data rate, at least 318 cases were ultimately required. The present study actually enrolled 563 cases, which met this requirement.

### 2.5 Analysis of influencing factors

Variables with *P* < 0.05 in univariate analysis were entered into LASSO regression. The optimal penalty parameter *λ* (lambda.1se) was determined using 10-fold cross-validation to screen for key predictive variables. Variables selected by LASSO were then subjected to multicollinearity diagnosis, and those with a variance inflation factor (VIF) > 10 were excluded. The final set of variables was incorporated into a multivariable logistic regression model (coding standard: no = 0, yes = 1) to identify independent risk factors for POSD.

### 2.6 Model development and performance evaluation

Traditional logistic regression and seven machine learning algorithms (random forest, XGBoost, naive Bayes, decision tree, support vector machine, k-nearest neighbors, and artificial neural network) were used to develop prediction models for POSD. Internal validation was performed using the independent validation set.

For the random forest model, hyperparameters were determined using grid search combined with 5-fold cross-validation.

Discrimination was assessed using the receiver operating characteristic (ROC) curve, area under the curve (AUC) with 95% confidence intervals, accuracy, sensitivity, specificity, precision, F1 score, and Youden's index. Calibration was evaluated using calibration curves and the Brier score. Clinical utility was assessed using decision curve analysis (DCA). For the best-performing model (random forest), SHAP analysis was performed on the validation set to provide post-hoc interpretability.

### 2.7 Statistical analysis

Statistical analyses were performed using SPSS 25.0 and R 4.5.1. Quantitative data were tested for normality using the Shapiro–Wilk test. Normally distributed data were expressed as mean ± standard deviation (SD), and non-normally distributed data as median (25th–75th percentile) [M(P25, P75)]. Categorical data were presented as frequency (percentage) [n (%)]. For between-group comparisons, the independent samples t-test was used for normally distributed quantitative data, the Mann–Whitney U test for non-normally distributed quantitative data, and the chi-square (*χ*^2^) test for categorical data. A two-sided significance level of *α* = 0.05 was adopted. For handling of missing data, continuous variables with a missing rate <5% were imputed using the median, and categorical variables with a missing rate <5% were imputed using the mode. Cases with any variable missing rate >20% were excluded from the analysis.

## 3 Results

### 3.1 Patient characteristics and incidence of POSD

A total of 563 patients were included, of whom 350 developed POSD (incidence 62.2%). In the training set (*n* = 338), 210 patients (62.1%) had POSD. Univariate analysis showed that nine variables were significantly associated with POSD (*P* < 0.05): drinking history, nocturia frequency, SII, blood transfusion, intraoperative hypotension, preoperative noise score, HADS-A score, HADS-D score, and moderate-to-severe cough-related pain (Table 1).

**Table 1:** Characteristics of the cohort.

Variables	Non-POSD	POSD	*P*-values
Demographics			
Age[years, M(P25, P75)]	74.00 (69.75,78.25)	73.00 (69.00,76.00)	0.445
BMI[kg/m2, M(P25, P75)]	23.33 (20.94,25.21)	22.98 (21.11,25.25)	0.656
Occupation, n(%)			
Farmer	49 (38.28)	93 (44.29)	0.518
Worker	29 (22.66)	46 (21.90)	
Other	50 (39.06)	71 (33.81)	
Education, n(%)			
Lower education	85 (66.41)	149 (70.96)	0.610
Advanced education	43 (33.59)	61 (29.04)	
Smoking history, n(%)	31 (24.22)	39 (18.57)	0.214
Drinking history, n(%)	18 (14.06)	48 (22.86)	0.048
Hypertension, n(%)	60 (46.88)	85 (40.48)	0.249
Diabetes, n(%)	15 (11.72)	26 (12.38)	0.856
Coronary heart disease, n(%)	17 (13.28)	19 (9.05)	0.221
Stroke, n(%)	14 (10.94)	15 (7.14)	0.227
Clinical data			
ASA classification, n(%)			
Level II	52 (40.62)	108 (51.43)	0.054
Level III	76 (59.38)	102 (48.57)	
Nocturia frequency, n(%)			
Mild (<3 times per night)	83 (64.84)	102 (48.57)	0.004
Moderate to severe (≥3 times per night)	45 (35.16)	108 (51.43)	
Opioid dose [mg, M(P25, P75)]	1.20 (1.10,1.30)	1.20 (1.15,1.25)	0.732
Use of propofol, n(%)	62 (48.44)	83 (39.52)	0.108
Use of sevoflurane, n(%)	16 (12.50)	38 (18.10)	0.173
Use of norepinephrine, n(%)	53 (41.41)	74 (35.24)	0.256
Surgery time [min, M(P25, P75)]	85.50 (55.75,119.25)	79.00 (55.00,110.75)	0.454
Anesthesia time [min, M(P25, P75)]	115.50 (92.50,147.75)	113.50 (88.00,146.75)	0.500
Extubation time [min, M(P25, P75)]	20.00 (10.00,37.00)	25.00 (15.00,35.00)	0.059
Fluid volume [mL,M(P25, P75)]	1,000.00 (600.00,1,100.00)	1,000.00 (600.00,1,100.00)	0.920
Blood transfusion, n(%)	5 (3.91)	25 (11.90)	0.002
Use of analgesic pump, n(%)	9 (7.03)	17 (8.10)	0.722
Intraoperative hypotension, n(%)	88 (68.75)	165 (78.57)	0.044
Preoperative noise score [points, M(P25, P75)]	66.00 (60.75,73.00)	69.00 (64.00,75.00)	0.018
Preoperative HADS-A score [points, M(P25, P75)]	3.00 (2.00,4.00)	4.00 (3.00,5.75)	<0.001
Preoperative HADS-D score [points, M(P25, P75)]	1.00 (0.00,2.00)	1.00 (1.00,2.00)	0.031
Preoperative VAS pain score at rest, n(%)			
None or mild (0–3 points)	127 (99.22)	206 (98.10)	0.715
Moderate to severe (>3 points)	1 (0.78)	4 (1.90)	
Preoperative cough-related pain, n(%)			
None or mild (0–3 points)	115 (89.84)	161 (76.67)	0.002
Moderate to severe (>3 points)	13 (10.16)	49 (23.33)	
Laboratory findings			
TPSA[ng/mL, M(P25, P75)]	6.28 (2.74,13.11)	5.96 (2.36,14.11)	0.744
FPSA[ng/mL, M(P25, P75)]	1.39 (0.64,2.89)	1.50 (0.55,2.98)	0.736
FPSA/TPSA	0.22 (0.17,0.30)	0.22 (0.17,0.30)	0.991
RBC count [10^12^/L, M(P25, P75)]	4.59 (4.12,5.06)	4.61 (4.16,4.96)	0.952
WBC count [10⁹/L, M(P25, P75)]	6.50 (5.55,8.12)	7.02 (5.80,8.54)	0.096
NLR	3.05 (2.14,4.50)	3.24 (2.24,5.10)	0.109
PLR	161.68 (126.77,208.93)	170.89 (131.60,253.06)	0.093
SII	662.89 (451.64,1,072.52)	823.40 (552.19,1,177.59)	0.005
CRP, n(%)			
Normal	121 (94.53)	202 (96.19)	0.472
Abnormal	7 (5.47)	8 (3.81)	
Total protein [g/L, M(P25, P75)]	68.16 (63.77,72.07)	69.00 (65.70,72.38)	0.158
Albumin [g/L, M(P25, P75)]	40.50 (37.70,43.15)	40.40 (38.15,42.59)	0.695
ALT[U/L, M(P25,P75)]	16.00 (13.00,24.00)	16.50 (12.25,24.75)	0.871
AST[U/L, M(P25,P75)]	20.00 (17.00,23.25)	21.00 (17.00,24.00)	0.545
Urea [mmol/L, M(P25, P75)]	6.29 (5.12,7.62)	5.90 (5.02,7.32)	0.290
Uric acid [*μ*mol/L, M(P25, P75)]	336.00 (275.75,390.25)	347.00 (289.75,415.00)	0.054
Creatinine [μmol/L, M(P25, P75)]	91.00 (77.50,103.00)	87.00 (76.25,99.00)	0.265

### 3.2 Variable selection and independent risk factors

The nine significant variables were entered into LASSO regression with lambda.1se, yielding eight variables with non-zero coefficients: drinking history, nocturia frequency, SII, blood transfusion, intraoperative hypotension, preoperative noise score, HADS-A score, and moderate-to-severe cough-related pain. Multivariable logistic regression analysis showed that preoperative HADS-A score, nocturia frequency (≥3 times/night), SII, preoperative noise score, and moderate-to-severe cough-related pain were independent risk factors for POSD (*P* < 0.05) (Table 2; Figures 1,2).

**Table 2:** Independent risk factors for postoperative sleep disturbance identified by logistic regression analysis.

Variable	*β* Coefficient	SE value	Wald *χ*2 value	OR(95%CI)	*P value*
Drinking history					
No	-	-	-	1.000 (1.000,1.000)	-
Yes	0.673	0.346	3.790	1.961 (0.995,3.862)	0.052
Preoperative HADS-A score	0.334	0.072	21.335	1.397 (1.212,1.610)	<0.001
Nocturia frequency, n(%)					
Mild (<3 times per night)	-	-	-	1.000 (1.000,1.000)	-
Moderate to severe (≥3 times per night)	0.601	0.253	5.618	1.824 (1.110,2.997)	0.018
SII					
Low level	-	-	-	1.000 (1.000,1.000)	-
High level	0.603	0.258	5.466	1.827 (1.102,3.029)	0.019
Blood transfusion					
No	-	-	-	1.000 (1.000,1.000)	-
Yes	0.783	0.572	1.874	2.188 (0.713,6.715)	0.171
Intraoperative hypotension					
No	-	-	-	1.000 (1.000,1.000)	-
Yes	0.306	0.287	1.139	1.358 (0.774,2.381)	0.286
Preoperative noise score	0.025	0.012	4.326	1.026 (1.001,1.051)	0.038
Preoperative cough-related pain					
None or mild (0–3 points)	-	-	-	1.000 (1.000,1.000)	-
Moderate to severe (>3 points)	1.108	0.371	8.903	3.027 (1.462,6.266)	0.003

OR, odds ratio; 95% CI, 95% confidence interval; SII, Systemic Immune-Inflammation Index.

**Figure 1:**
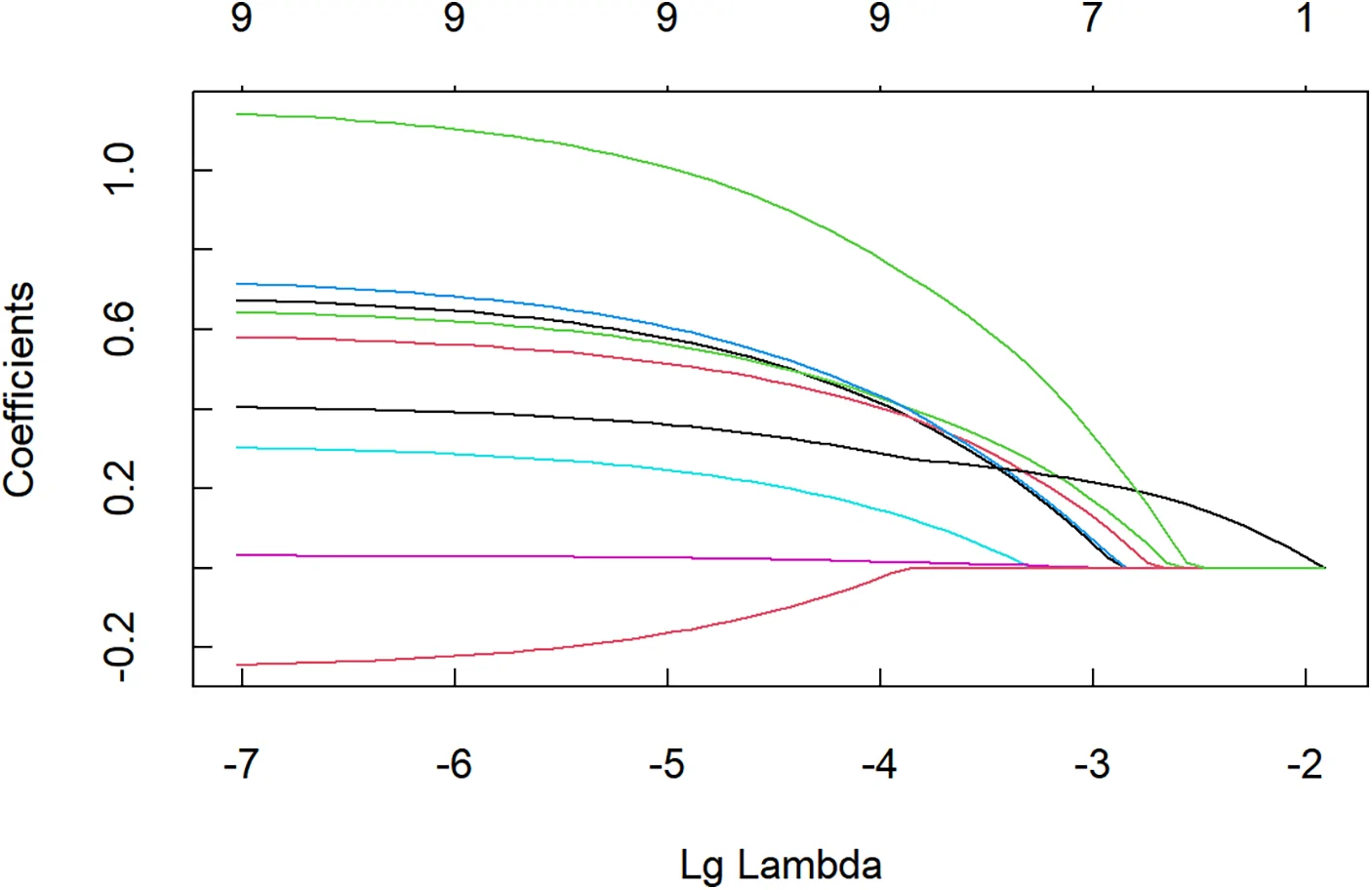
Variable selection process plot of risk factors for postoperative sleep disturbance via LASSO regression.

**Figure 2:**
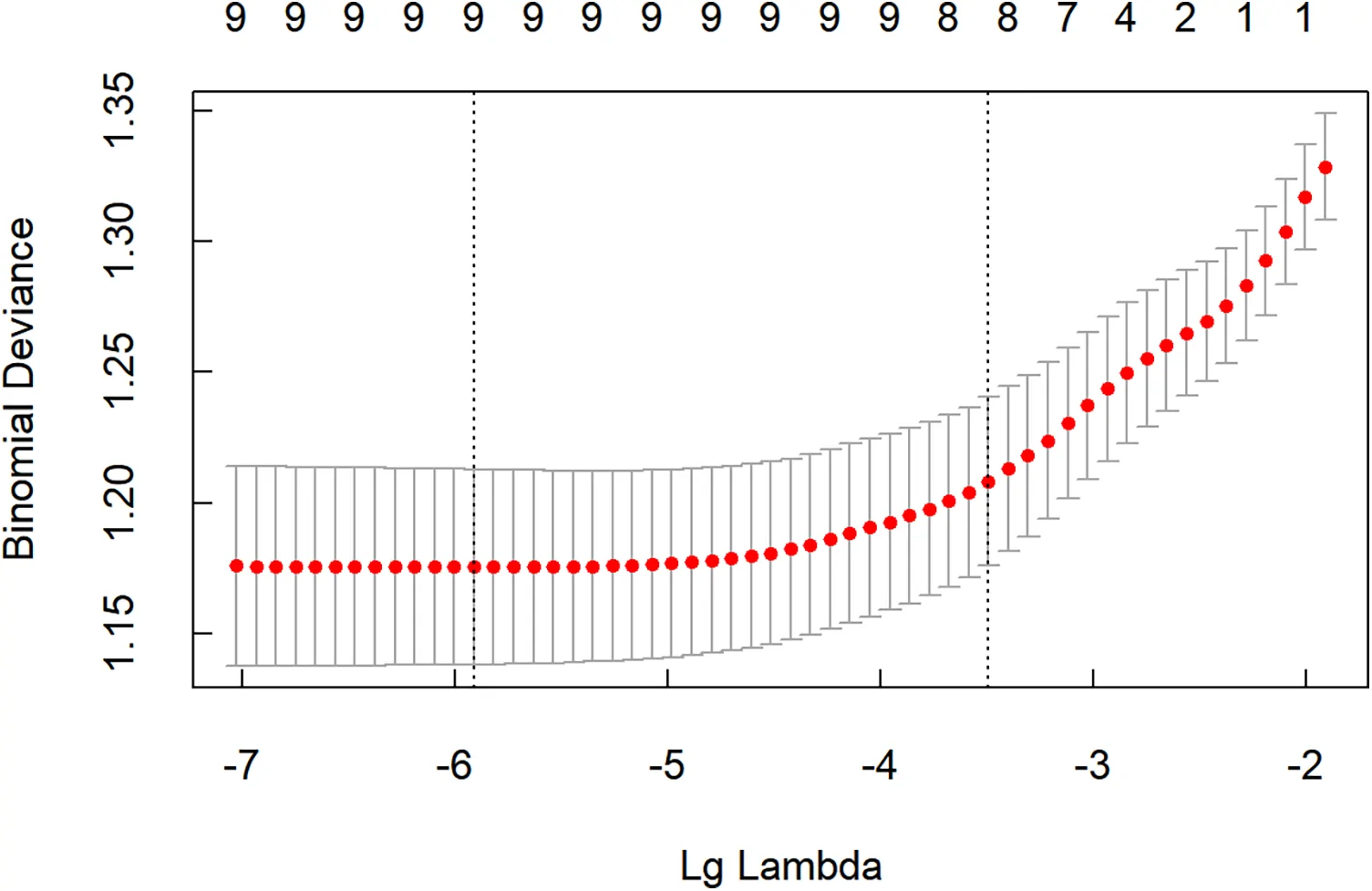
Variable selection process for risk factors of postoperative sleep disturbance via LASSO regression with 10-fold cross-validation.

### 3.3 Model performance comparison

The random forest model achieved the best overall performance in both the training set (AUC = 0.926, 95% CI: 0.89–0.96) and the validation set (AUC = 0.964, 95% CI: 0.93–0.99), with higher accuracy, sensitivity, and F1 score than the other models. The logistic regression model achieved a training set AUC of 0.749 (95% CI: 0.69–0.81) and a validation set AUC of 0.737 (95% CI: 0.67–0.80) (Tables 3,4; Figures 3,4). The higher AUC in the validation set may be due to random sampling variability or a more homogeneous subset.

**Table 3:** Performance evaluation of machine learning algorithms and logistic regression model in the training set.

Model	AUC	Accuracy	Sensitivity	Specificity	Precision	F1-score	Youden's index
Random_Forest	0.926	0.840	0.905	0.734	0.848	0.876	0.716
XGBoost	0.860	0.769	0.886	0.578	0.775	0.827	0.563
ANN	0.780	0.725	0.833	0.547	0.751	0.790	0.415
SVM	0.768	0.707	0.891	0.406	0.711	0.791	0.429
Logistic_Regression	0.749	0.669	0.576	0.820	0.840	0.684	0.397
Naive_Bayes	0.742	0.689	0.757	0.578	0.747	0.752	0.384
KNN	0.740	0.707	0.857	0.461	0.723	0.784	0.356
Decision_Tree	0.646	0.692	0.838	0.453	0.715	0.772	0.291

**Table 4:** Performance evaluation of machine learning algorithms and logistic regression model in the validation set.

Model	AUC	Accuracy	Sensitivity	Specificity	Precision	F1-score	Youden's index
Random_Forest	0.964	0.880	0.964	0.741	0.860	0.909	0.798
XGBoost	0.913	0.840	0.929	0.694	0.833	0.878	0.657
ANN	0.841	0.782	0.864	0.647	0.801	0.832	0.511
Decision_Tree	0.817	0.760	0.893	0.541	0.762	0.822	0.464
Naive_Bayes	0.811	0.724	0.864	0.494	0.738	0.796	0.469
SVM	0.781	0.720	0.843	0.518	0.742	0.789	0.479
KNN	0.778	0.693	0.800	0.518	0.732	0.765	0.429
Logistic_Regression	0.737	0.676	0.814	0.447	0.708	0.758	0.371

**Figure 3:**
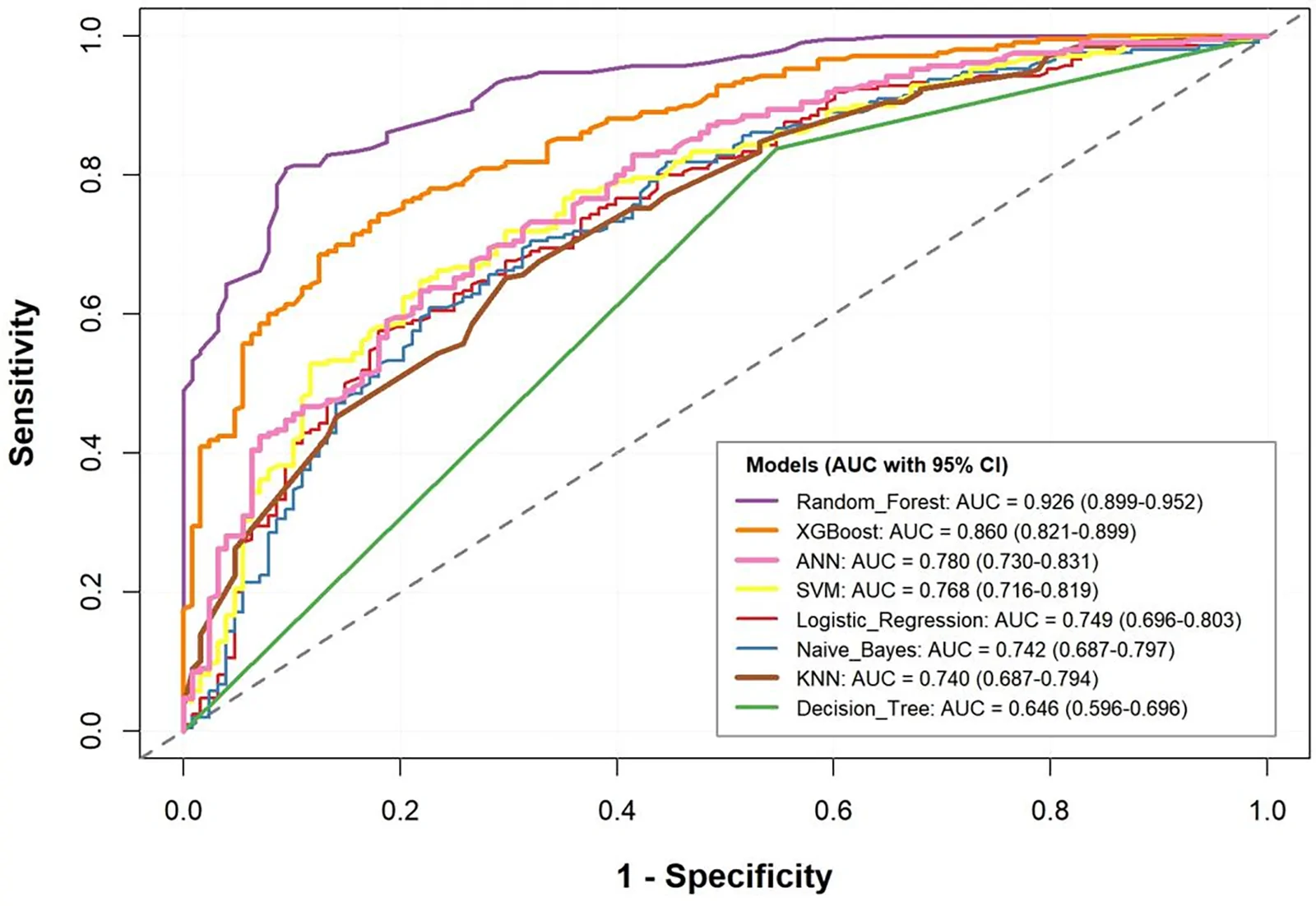
ROC and AUC of machine learning algorithms and logistic regression model for predicting POSD in the training set.

**Figure 4:**
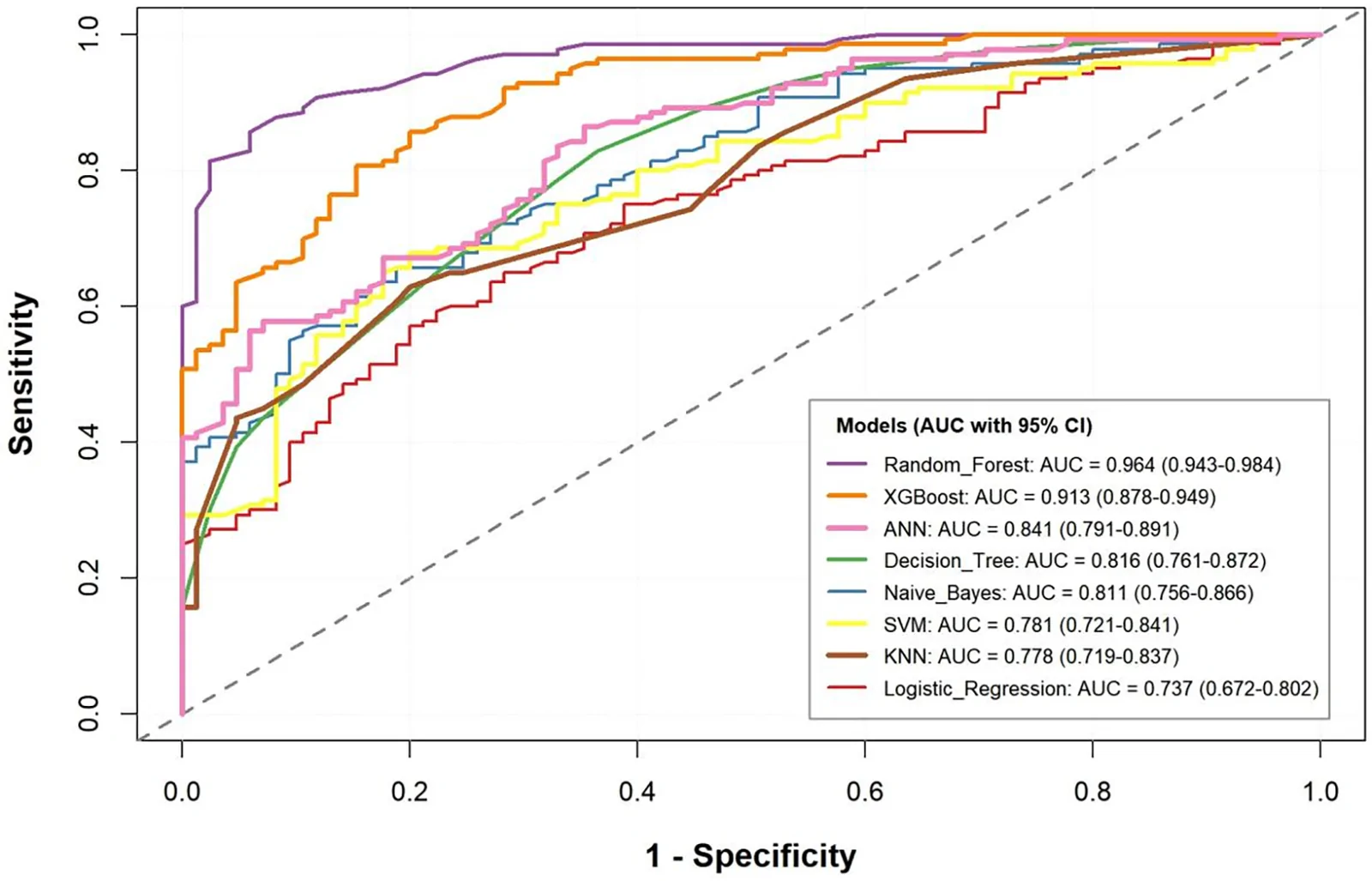
ROC and AUC of machine learning algorithms and logistic regression model for predicting POSD in the validation set.

Calibration curves showed good agreement between predicted and observed probabilities for the random forest model (Brier score: 0.106 in the training set, 0.092 in the validation set). Decision curve analysis demonstrated that the random forest model provided the highest net clinical benefit across threshold probability ranges of 0.08–0.99 (training) and 0.10–0.99 (validation) (Figures 5–8).

**Figure 5:**
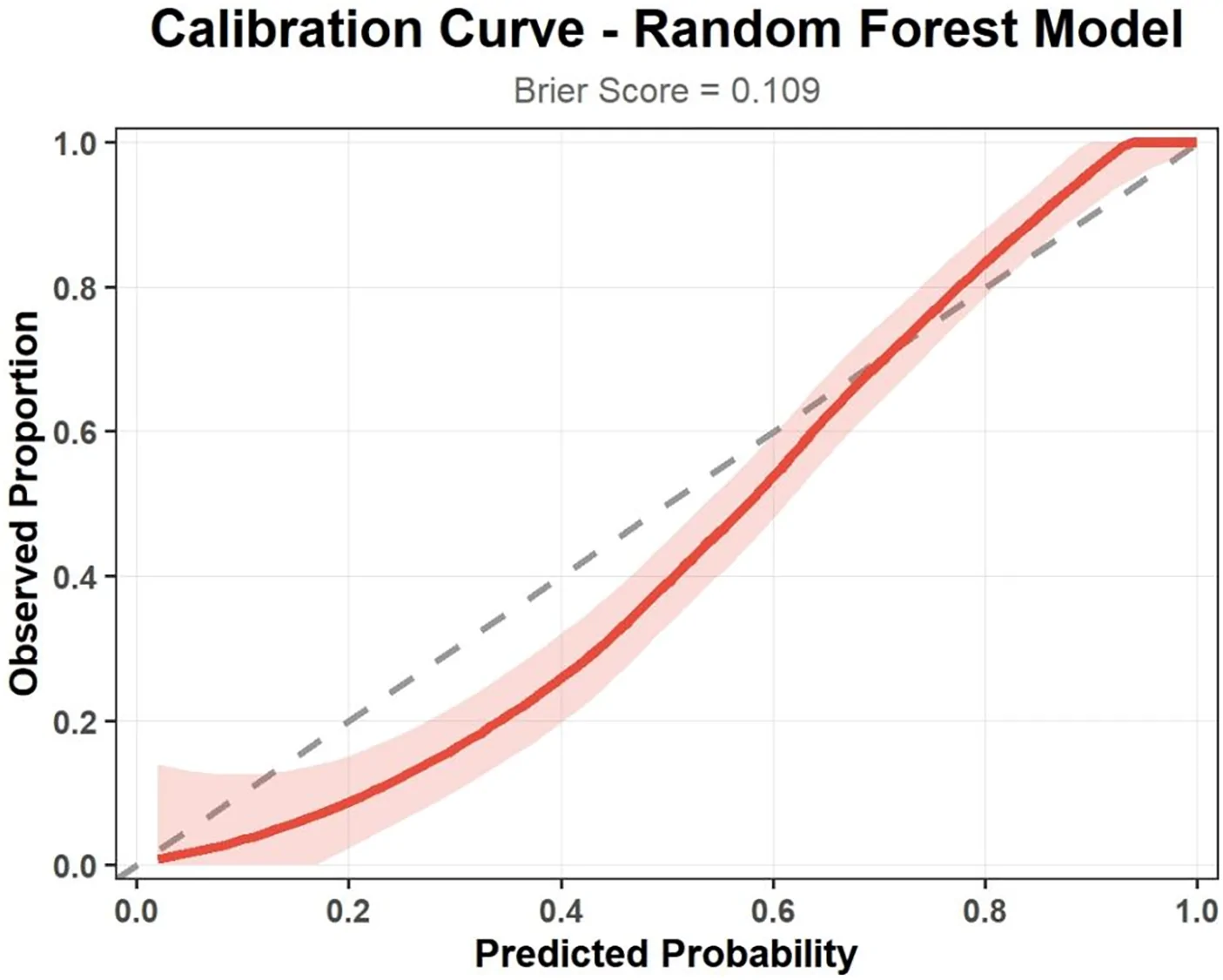
Calibration curve of the random forest prediction model in the training set.

**Figure 6:**
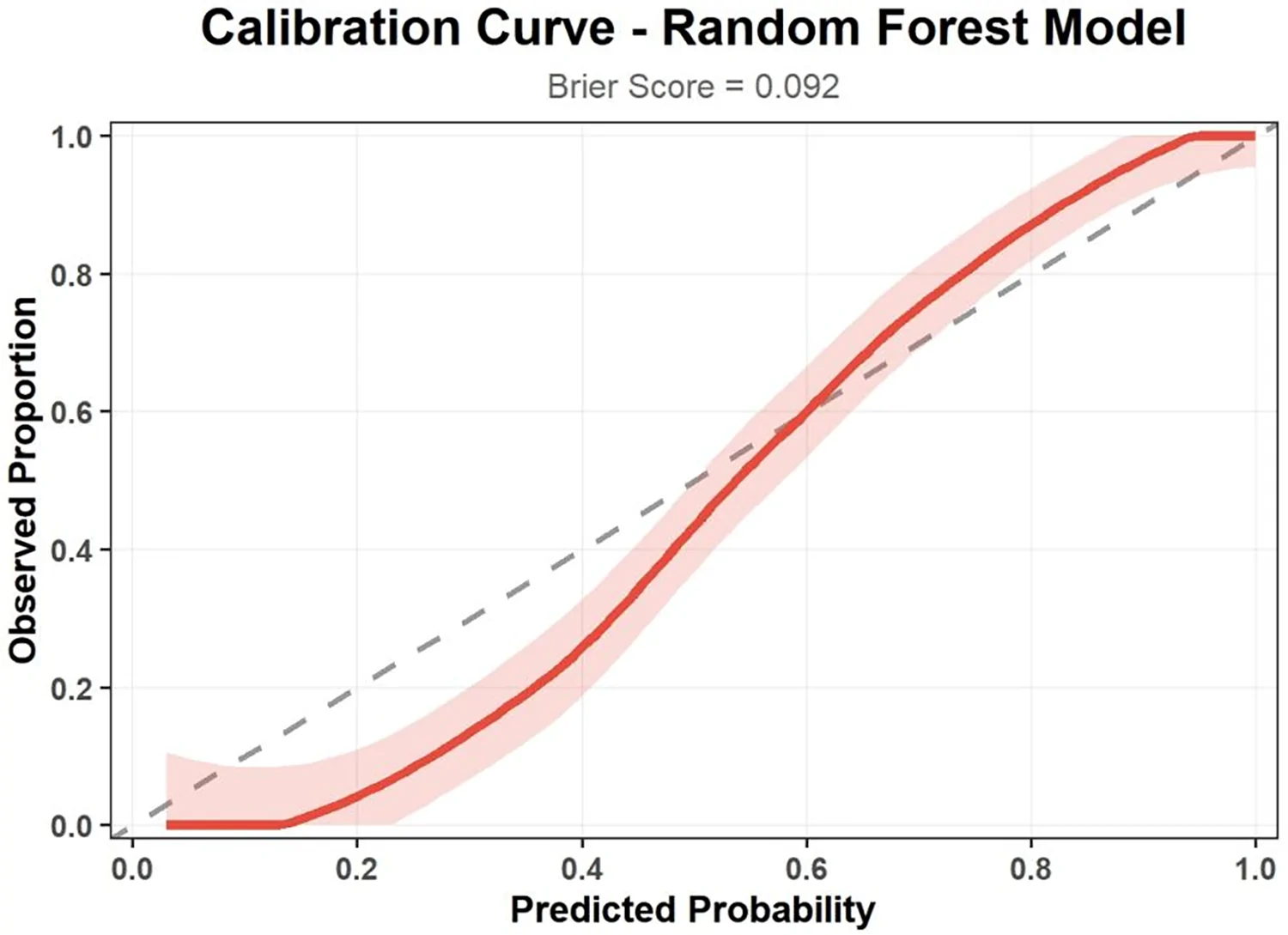
Calibration curve of the random forest prediction model in the validation set.

**Figure 7:**
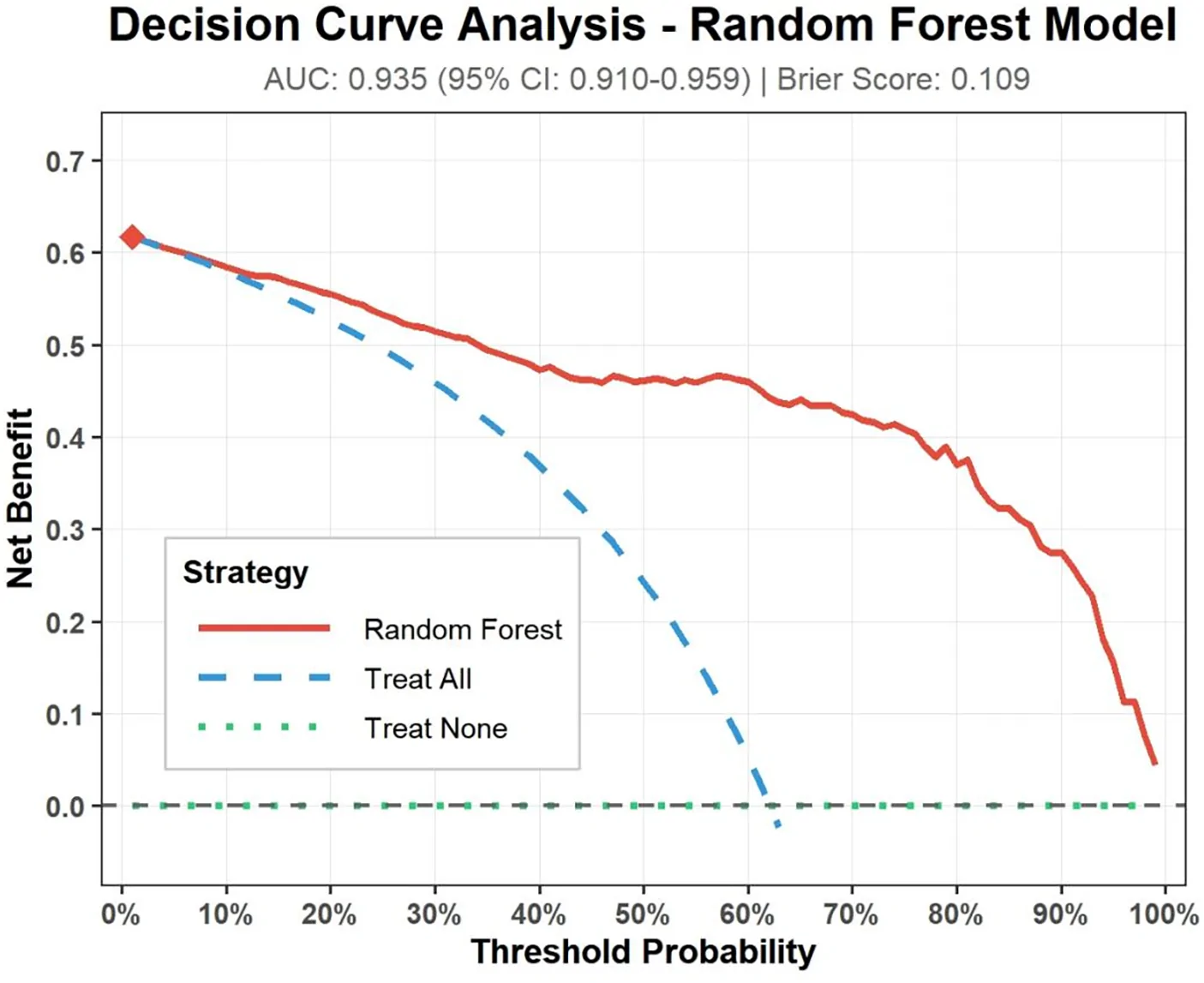
Decision curve of the random forest model in the training set.

**Figure 8:**
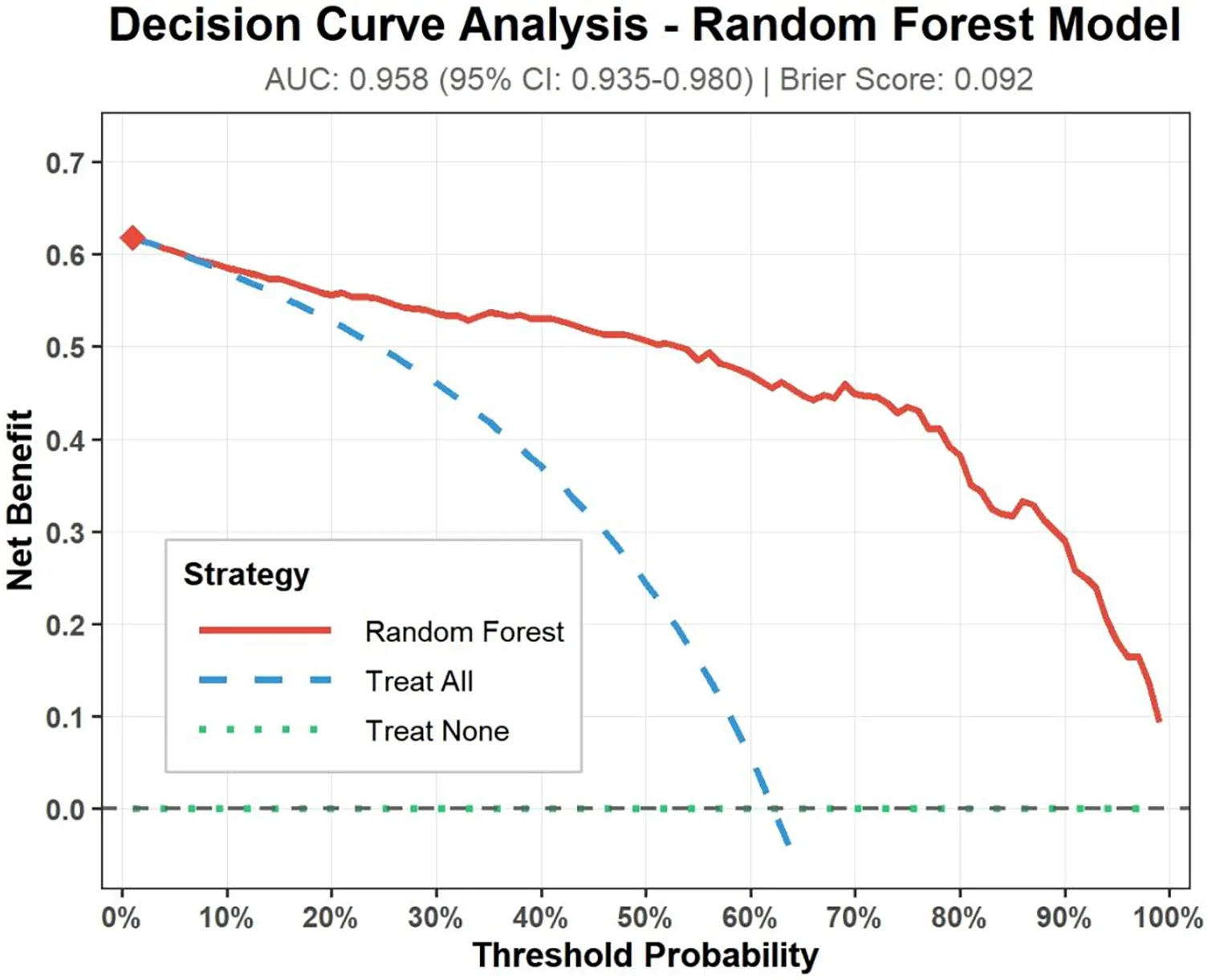
Decision curve of the random forest model in the validation set.

### 3.4 SHAP interpretation of the random forest model

SHAP feature importance ranking showed that HADS-A score contributed the most to the predictions, followed by nocturia frequency, SII, noise score, and moderate-to-severe cough-related pain (Figure 9). The SHAP beeswarm plot revealed that the SHAP values of HADS-A score had the widest distribution, indicating that anxiety status contributed most significantly to the model predictions (Figure 10).

**Figure 9:**
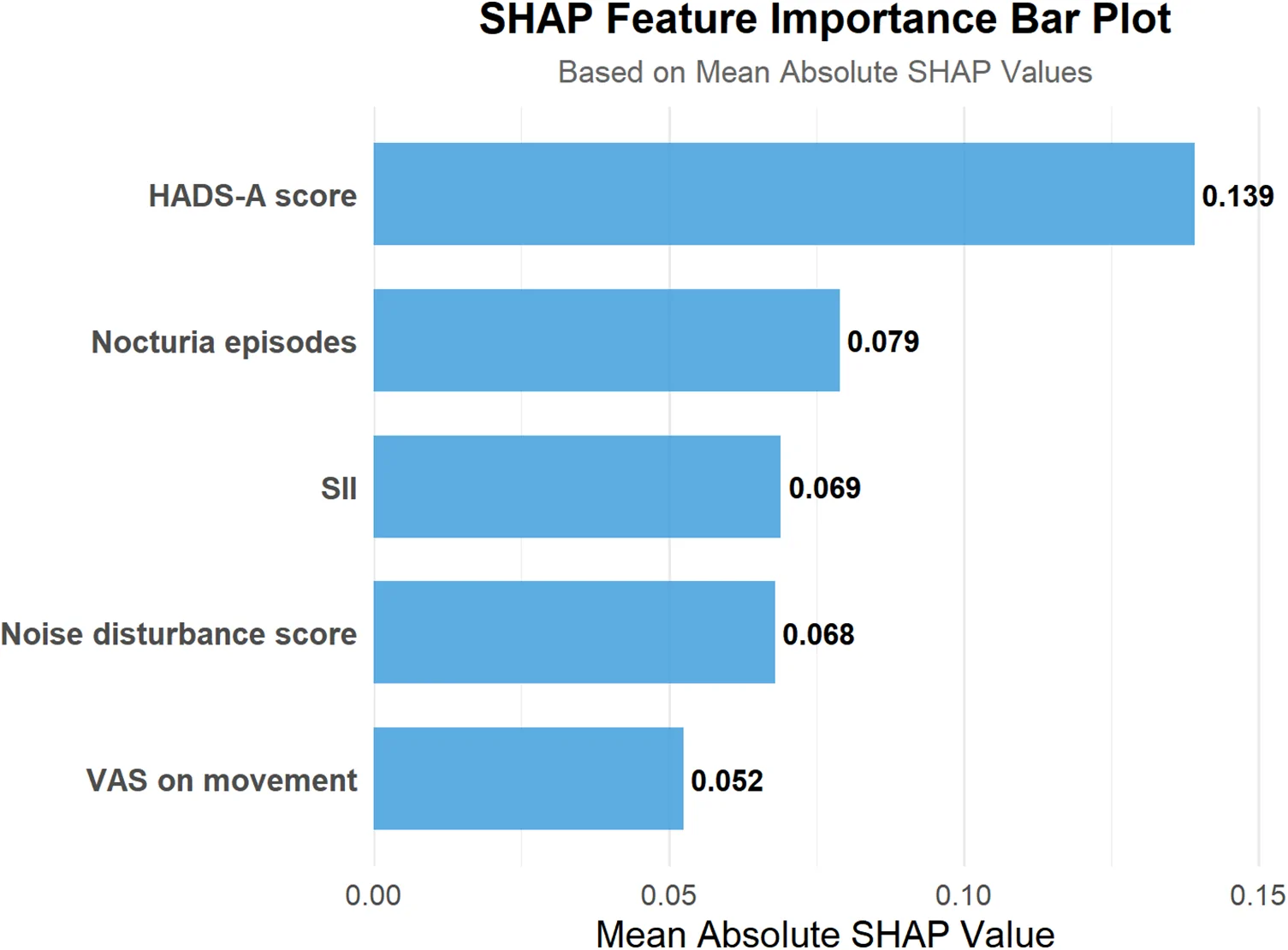
Interpretation of feature importance using SHAP values for the random forest model.

**Figure 10:**
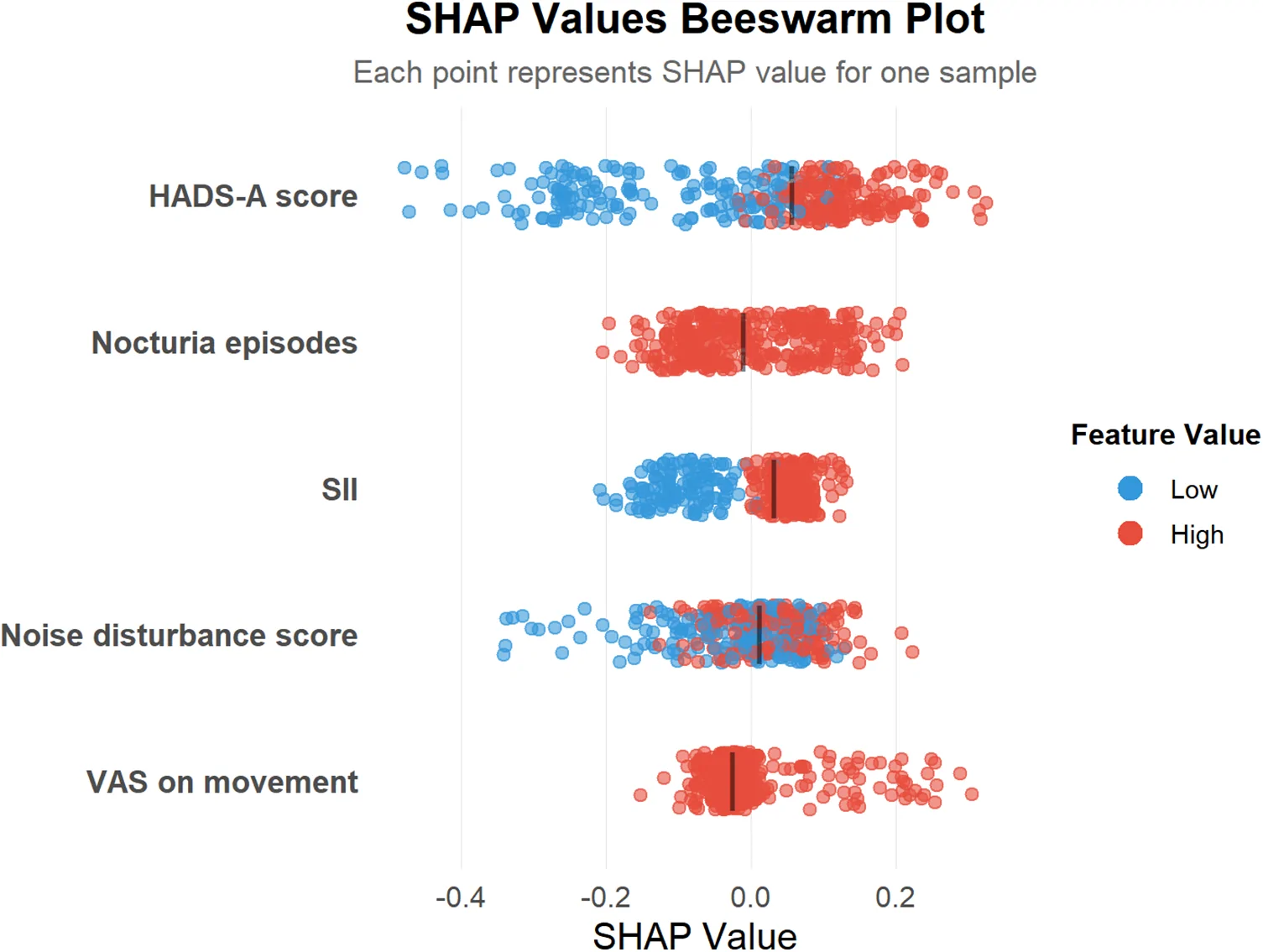
SHAP beeswarm plot of the random forest model.

## 4 Discussion

This study found that the incidence of POSD in elderly patients undergoing transurethral prostate surgery was 62.2%, which is generally consistent with previous reports (10). Preoperative anxiety, nocturia frequency (≥3 times/night), elevated SII, high noise score, and moderate-to-severe cough-related pain were identified as independent risk factors. The random forest model showed significantly better predictive performance than logistic regression and the other six machine learning models, and SHAP analysis enhanced its interpretability.

Preoperative anxiety was the strongest predictor of POSD, consistent with findings in total hip arthroplasty and spinal surgery (15, 16). A systematic review indicated that preoperative sleep disturbance and anxiety are two core risk factors for POSD (10). Anxiety activates the hypothalamic-pituitary-adrenal (HPA) axis and sympathetic nervous system, promotes cortisol release, interferes with the GABAergic system, and increases arousal levels (17).

Even though indwelling catheters eliminate the actual need to urinate after surgery, preoperative nocturia frequency remained a stable predictor, suggesting a “conditioned arousal” pattern. The proposed mechanism involves repeated association of bladder fullness sensation with sleep environment cues, leading to long-term sensitization of the “bladder–brain axis” (insula, anterior cingulate cortex) (18). Clinical studies confirm that nocturia ≥3 times/night is directly associated with poor sleep quality (19).

In this study, ROC curve analysis determined that the optimal cutoff value of SII for predicting POSD was 603.98. This value not only quantifies the risk threshold of immune-inflammatory burden—indicating that patients with a preoperative SII >603.98 have a significantly higher risk of POSD—but also provides a simple and objective risk stratification tool for clinical practice, facilitating preoperative identification of high-risk individuals and targeted interventions. The obtained cutoff value is generally consistent with the findings of several previous studies (20, 21). As an integrated inflammatory marker, SII showed a stronger association with POSD than NLR or PLR (22, 23). Surgical stress elevates SII and induces sleep disturbance, while sleep disturbance sustains inflammation via persistent activation of the HPA axis and sympathetic nervous system (24, 25). Furthermore, elevated SII has been reported as a biomarker for POSD in elderly patients undergoing joint replacement (26).

The bidirectional relationship between pain and sleep disturbance is well established: pain impairs sleep, and sleep deficiency lowers pain threshold (27). Mechanisms include decreased dopaminergic activity (28, 29), serotoninergic dysregulation (30, 31), HPA axis rhythm disruption (32, 33), and release of inflammatory mediators (34). In our study, patients often had preoperative lower urinary tract symptoms (frequency, urgency, dysuria) or even preoperative catheterization, all of which can be sources of pain.

To our knowledge, this is the first study to compare logistic regression with seven machine learning algorithms for predicting POSD in this specific population and to introduce SHAP interpretability. Most existing POSD prediction studies rely on traditional logistic regression (35), whereas machine learning can handle non-linear relationships and extract key features from high-dimensional data. The random forest model exhibited the best discriminative ability, sensitivity, and calibration. Its advantage stems from bagging ensemble learning and random feature selection, which capture non-linear interactions and resist overfitting (36). Our findings align with a multicenter survey (37), confirming the advantage of ensemble learning in complex biomedical data. SHAP analysis clarified the direction and magnitude of each predictor's contribution, enhancing clinical acceptability (38, 39). The prediction model provides a quantitative basis for developing targeted intervention strategies (e.g., preoperative psychological counseling for patients with high anxiety, optimization of the acoustic environment for those with high noise exposure), significantly improving the integration of machine learning models with the practical needs of perioperative clinical care.

### 4.1 Limitations

This study has several limitations. First, POSD was assessed mainly using subjective patient-reported scales rather than objective measures such as polysomnography (PSG), electroencephalography (EEG), or actigraphy. Continuous objective sleep monitoring may improve assessment accuracy in the future. Second, this was a single-center study without external validation, which limits its generalizability. Third, the validation set AUC (0.964) was higher than the training set AUC (0.926), possibly reflecting distributional differences between the two sets or the relatively small size of the validation set rather than overfitting. Although LASSO with lambda.1se was used to reduce overfitting, external validation remains essential. Fourth, patients with pre-existing sleep disturbance were excluded; this clinically important subgroup should be included in future stratified analyses.

## 5 Conclusion

The incidence of POSD in elderly patients undergoing transurethral prostate surgery is 62.2%. Preoperative anxiety, increased nocturia (≥3 times/night), elevated SII, high noise score, and moderate-to-severe cough-related pain are independent risk factors. The random forest prediction model demonstrates optimal discriminative ability, calibration, and clinical utility, with good robustness and interpretability. Large-scale, multicenter studies are needed to further validate its generalizability and clinical applicability.

## Data Availability

The original contributions presented in the study are included in the article/supplementary material, further inquiries can be directed to the corresponding author.
